# Supplementary material for: Workflow for detecting biomedical articles with underlying open and restricted-access datasets
Source: PLoS One. 2024 May 8;19(5):e0302787. doi: 10.1371/journal.pone.0302787 (PMC11078384; doi:10.1371/journal.pone.0302787)
Supplement: S1 File — Repositories with less than three datasets are not displayed. (PDF) [file pone.0302787.s001.pdf]

S1 Table. Number of datasets per repository for manually confirmed open data cases in 2021. Repositories with less than three datasets are not displayed.

	repository	type	n
1	Gene Expression Omnibus (GEO)	Disciplinary	106
2	Figshare	General-purpose	63
3	Open Science Framework (OSF)	General-purpose	46
4	Zenodo	General-purpose	39
5	PRoteomics IDEntifications Database	Disciplinary	32
6	NCBI Sequence Read Archive	Disciplinary	30
7	NCBI Nucleotide	Disciplinary	22
8	GitHub	General-purpose	21
9	European Nucleotide Archive (ENA)	Disciplinary	19
10	ArrayExpress	Disciplinary	14
11	Worldwide Protein Data Bank (wwPDB)	Disciplinary	11
12	Mendeley Data	General-purpose	10
13	Electron Microscopy Data Bank (EMDB)	Disciplinary	7
14	Mass Spectrometry Interactive Virtual Environment (MassIVE)	Disciplinary	7
15	Genome-wide Association Studies Catalog (GWAS Catalog)	Disciplinary	6
16	Dryad	General-purpose	3
17	GenBank	Disciplinary	3
