# Supplementary figures and images for: Workflow for detecting biomedical articles with underlying open and restricted-access datasets
Source: PLoS One. 2024 May 8;19(5):e0302787. doi: 10.1371/journal.pone.0302787 (PMC11078384; doi:10.1371/journal.pone.0302787)

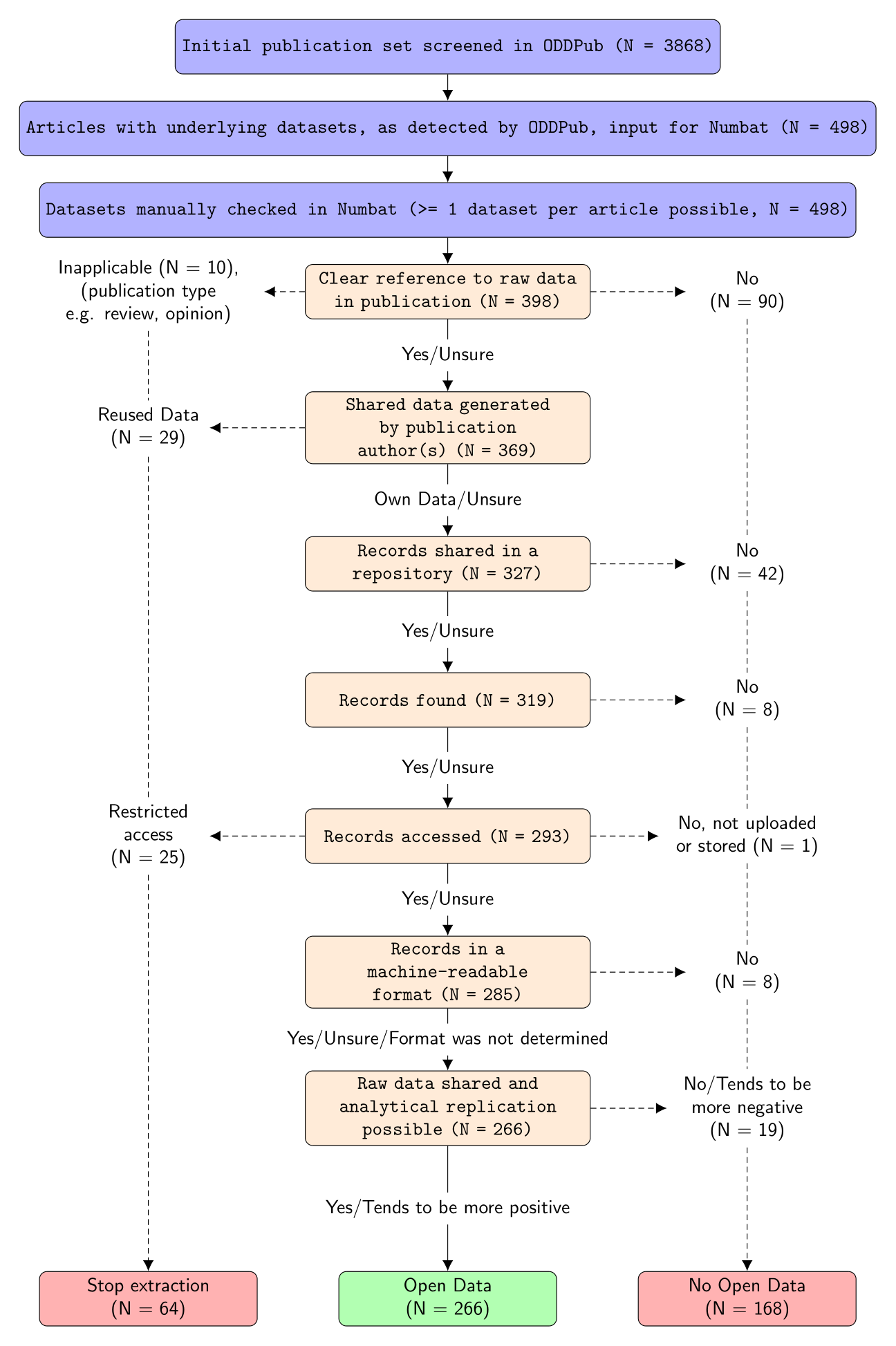

Supplement: S1 Fig — Numbers in beige boxes indicate the number of articles screened at the respective stage which complied with the criterion in question. Please note that unlike in Fig 1 und S2 Fig, the numbers refer to an earlier version of the extraction workflow in which we extracted only one dataset per article. Thus, the number of datasets and of articles is identical in this case. (TIFF) [file pone.0302787.s002.tiff]

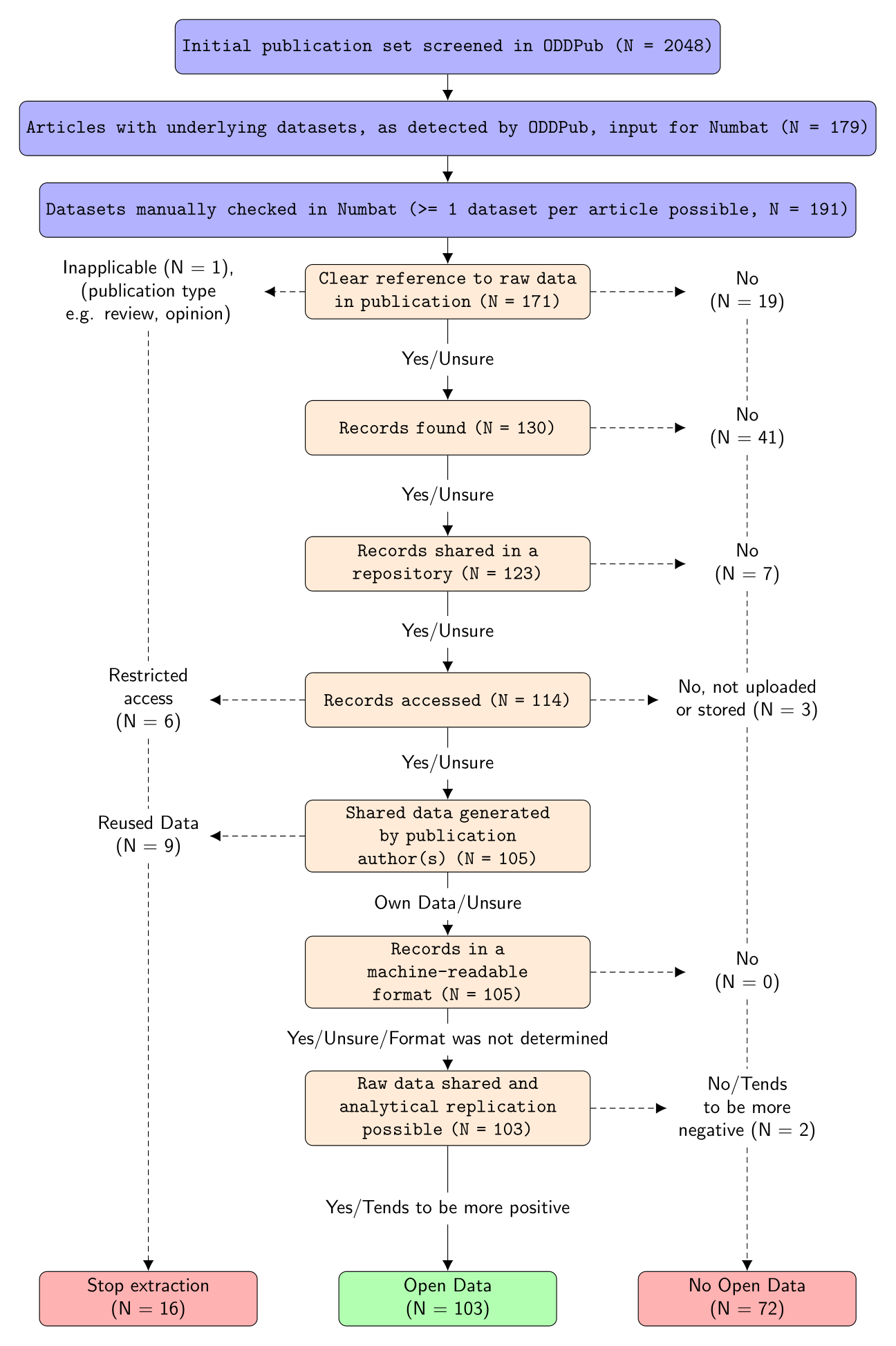

Supplement: S2 Fig — Numbers in beige boxes indicate the number of articles screened at the respective stage which complied with the criterion in question. (TIFF) [file pone.0302787.s003.tiff]
