# Supplementary material for: Workflow for detecting biomedical articles with underlying open and restricted-access datasets
Source: PLoS One. 2024 May 8;19(5):e0302787. doi: 10.1371/journal.pone.0302787 (PMC11078384; doi:10.1371/journal.pone.0302787)
Supplement: Attachment — Submitted filename: Response to reviewers.pdf [file pone.0302787.s004.pdf]

Dear PLOS ONE Editorial Team, der Reviewers,

please find below our answers to the points raised.

Best regards,

Evgeny Bobrov (in the name of all authors)

Journal Requirements:

When submitting your revision, we need you to address these additional requirements.

1. Please ensure that your manuscript meets PLOS ONE's style requirements, including those for file naming. The PLOS ONE style templates can be found at https://journals.plos.org/plosone/s/file?id=wjVg/PLOSOne_formatting_sample_main_body.pdf and https://journals.plos.org/plosone/s/file?id=ba62/PLOSOne_formatting_sample_title_authors_affiliations.pdf

We have made several changes to comply with PLOS ONE's style requirements, including the naming of all figures. Also see below under point (3.).

2. We suggest you thoroughly copyedit your manuscript for language usage, spelling, and grammar. If you do not know anyone who can help you do this, you may wish to consider employing a professional scientific editing service.

Whilst you may use any professional scientific editing service of your choice, PLOS has partnered with both American Journal Experts (AJE) and Editage to provide discounted services to PLOS authors. Both organizations have experience helping authors meet PLOS guidelines and can provide language editing, translation, manuscript formatting, and figure formatting to ensure your manuscript meets our submission guidelines. To take advantage of our partnership with AJE, visit the AJE website (http://learn.aje.com/plos/) for a 15% discount off AJE services. To take advantage of our partnership with Editage, visit the Editage website (www.editage.com) and enter referral code PLOSEDIT for a 15% discount off Editage services. If the PLOS editorial team finds any language issues in text that either AJE or Editage has edited, the service provider will re-edit the text for free.

Upon resubmission, please provide the following:

The name of the colleague or the details of the professional service that edited your manuscript

We assume that this is a text which all authors receive and is not directed specifically to us. As no issues regarding language, spelling, and grammar were highlighted in the reviews, we have not taken any further action here.

A copy of your manuscript showing your changes by either highlighting them or using track changes (uploaded as a *supporting information* file)

A clean copy of the edited manuscript (uploaded as the new *manuscript* file)"

In addition to a clean copy of the edited version of the manuscript, we will also upload a version where the changes are tracked, except for three types of changes, which would have been made the version with tracked changes very difficult to follow:

(i) Reformatting of the in-text references according to PLOS ONE guidelines

(ii) Changes to the references themselves, including e.g. updated dates of resource retrieval; to highlight the newly added references, we set the font of added references green

(iii) Numbers in the text which changed due to a correction to the number of all articles screened by ODDPub (i.e., the denominator); also see below

3. Please include captions for your Supporting Information files at the end of your manuscript, and update any in-text citations to match accordingly. Please see our Supporting Information guidelines for more information: http://journals.plos.org/plosone/s/supporting-information.

We have included captions to our Supplementary Information files in the last section of the manuscript, and have places the caption of Fig. 1 at the appropriate location in the text, following the PLOS ONE guidelines for the descriptions of Figures and Tables. The in-text citations were updated as well.

Importantly, some numbers in the manuscript which relate to the Charité 2021 screening have slightly changed, as we corrected the number of article which were screened with ODDPub overall. We had reported this number to be 5044, but indeed it is 5119. This does not change any conclusions, and largely led to changes in the first decimal of percentages. The difference of 75 articles is due to a discrepancy between our dashboard and the article set reported here. We had based our calculations on the number in the dashboard, but closer scrutiny showed that the sample is not fully identical, due to different time points at which the samples were drawn from publication databases. If the reviewers do not receive this section of the response letter, we ask you to copy this section into the reviewer answers, as this is also of relevance to them.

Additional Editor Comments:

Please take particular care to the comments raised by all the reviewers (especially R3).

The technical contribution looks not thorough enough. The justification and motivations, along with the objectives of the work should be better elaborated and clearly articulated.

The methodology would require further, deeper investigation. Explain better and motivate the employed dataset and features. The reported computational evaluation and discussion show to be not robust enough, it should be articulated more.

Unfortunately the manuscript fails to meet the PLOS ONE publication criteria in its current form.

We have addressed the reviewer comments, especially regarding the methodology. Please find the individual comment below. We hope that taken together, the comments are sufficiently addressed and the manuscript can be accepted for publication.

Reviewers' comments:

Reviewer #1: The authors do not mention the journals from which they extract the DAS, I would recommend that they do so, to see which journals use this statement, as well as the repositories they have used to verify the presence or not of the data.

With regard to repositories, we have added an analysis of the repositories in which datasets underlying publications from 2021 have been shared. The table has been added as S1 Table. Three datasets were excluded to avoid double-dipping, as each of them had been underlying the analysis in two articles published in the same validation period. Importantly, we only extracted one dataset per article and repository. Thus, the numbers in the table do not

correspond to the overall number of datasets deposited. To extract this would not only have been much more time-consuming, but would require a more detailed operationalization of what a dataset is. Despite this limitation, the numbers indicate which repositories are commonly used in biomedical research, and indicate a dominance of repositories for genetic data, as already visualized in our FAIR Data Dashboard.

With regard to journals, the information which journal provided DAS and which didn't is not available to us. We screened article whole texts for statements indicative of data availability, and did not restrict ourselves to the DAS. We also did not specifically document whether a DAS was present. This is in line with our statement in the article that extracting DAS could be a future improvement to the algorithm, and indeed, the newest version of the algorithms approaches that question.

On page 18 it says Haven et al, but they do not add the year. On the reference list the year is 2022, but throughout the text it says 2023, is there a missing reference or is it a mistake?

Thank you for pointing this out. There was a mistake as we still listed the preprint from 2022 in the reference list, but in the meanwhile a reviewed article was published in 2023. The reference is now to the 2023 reviewed article.

Reviewer #2: The topic of the article is very interesting, important and applicable in current academic system.
The methodology workflow and the results are described clearly, systematically, in detail, and the research seems reproducible. However, since everything else is described in such detail, I suggest you add a few sentences about the protocol for the manual confirmation step. For example, you say "we checked, amongst other criteria, whether the dataset could be found" - how was this checked - using what software, which search terms? Are the raters' physicians or information specialists? This could affect both the results and the time needed for rating...

We have provided a complete list of the individual steps in the extraction, and we refer to the figures where this is shown. We have also added some information on the professional background of the raters. Details on the manual checking are described in an open protocol (Iarkaeva et al., 2022), to which we already refer in this paragraph, and thus, to keep it readable, we have kept the additional text short.

I tried to access the data using DOI link provided in Data availability statement, but did not gain access. Will this be open once the article is published?

Having the data available online is a matter of course for us, and the dataset was already online at the time of submission. Unfortunately, while the DOI was correct, something went wrong with the link, thank you for pointing this out.

I have two additional questions that don't need to be answered in this article, but can serve as "food for taught":
- Have you considered using other tools which you mention in the discussion on the same set of publications? From the introductory part, it's clear that the results would not be the same, because the methodology and definitions (hence the goals) are different, but it would still be interesting to compare the results and see if your tools and protocols missed anything.

This is also of high interest to us, and we would definitely like to conduct such an analysis. We are particularly interested in the tool DataStet, used to create both the French Open Science Monitor and the PLOS open science indicators dataset. The first step would probably

be to apply DataStet to Charité publications and compare the outcome with our results. Indeed, we consider doing so this year, but this would be out of scope of the present article.

- It would be interesting to compare presence of dataset statements, the actual datasets availability and their accessibility between OA articles and paywalled articles.

We appreciate the suggestion to compare the rates of data sharing for open access and paid access articles, and have added the analysis to the article. As to be expected, sharing data is more common for open access articles, which have a 9.5% probability of having manually confirmed open or restricted-access data, as opposed to 4.8% for paywalled articles. Importanly, this analysis only applies to the articles which we downloaded and screened with ODDPub at the time of validation (i.e., in 2022). For all articles of the Charité, this can only serve as an approximation, as we screened a large majority of Charité articles (80.0%), but not all. Articles were not screened for one or more of the following reasons (as also listed below in an answer to reviewer #3): (i) lacking institutional access to certain paid-access journals, (ii) failure to automatedly download open access articles for technical reasons, often due to barriers against automated download placed by the journals, and (iii) deliberate exclusion of some article types as e.g. obituaries and corrigenda to reduce the computational workload. While excluded article types should indeed not be part of this analysis, the other two cases should be in the sample to make a definitive statement about the share of available data by article type.

With respect to the other analyses suggested, we are not fully sure what is meant. If "presence of dataset statements" refers to statements as detected by ODDPub, this would only add the false positive cases which we manually removed. And if "accessibility" refers to one of the steps during the checking process, the same question could then be asked about all other extraction steps as well, e.g. "findability" and "deposition in a repository". As this is mentioned as "food for thought" we assume that this is not an issue that needs to be resolved, but if it is a point important to address, we would ask the reviewer to provide more detail on this suggestion.

Reviewer #3: The article presents a workflow to identify dataset mentions in scholarly articles that are publicly accessible. To this end, the authors employ existing (or elsewhere introduced) software packages and review the output manually. In particular, ODDPub (developed in the direct environment of the authors) is employed to identify dataset mentions from fulltext pdfs. Numbat (a tool to support systematic reviews) is used then to review the positive decisions of ODDPub. The authors estimate the IRR between 2 and 3 raters on sets of 100 resp. 20 publication and estimate a high reliability. The authors conclude that this manual curation process can be used to estimate the amount of mentions to publicly accessible datasets, but also that the set of publications should not be larger than the sample size used in the paper (~6000).

I appreciate the idea of identifying dataset mentions automatically to provide extra funding for "Open Scientists". However, I see some problems with the work at hand that should be handled before acceptance.

- From the original work on ODDPub the authors know that the Identification performance is very high. However, the authors note that there are some differences between the evaluation set and the set of articles at hand. I would like to see a more detailed evaluation including articles (at least a sub-sample) that have been sorted out by ODDPub.

We agree that since some time has passed and we introduced some changes, an extensive validation including a screen of a set of articles **not** flagged as open data articles by ODDPub would be ideal. However, given that the changes we introduced are relatively minor, we think that it is justified to still refer primarily to the validation performed for the article on ODDPub (Riedel et al., 2020). At the same time, to assess whether the former validation at least still appears plausible, we have screened a small set of 100 articles manually using the same screening method we had used to validate the ODDPub algorithm for aforementioned publication. To do so, we searched in the fulltext for keywords and word roots like "data", "dataset", "access*", "availab*" etc. We found that ODDPub missed one restricted-access case. This suggests a false negative rate of 1%, which is lower than the 3.4% reported in Riedel et al. However, 17 out of 24 cases missed there were supplemental materials, which we now exclude. Thus, the numbers are not directly comparable, but excluding supplemental materials would have led to a rate of 1.0% in the Riedel et al. sample, which would be well in line with the value observed now. Of course, it must be acknowledged that 100 articles are a small sample for such a validation, given the low probability of false negatives, and thus the value of 1% we find now should only serve as supportive evidence in light with our much more extensive previous evaluation of 792 articles. We have added this in the methods section.

- For an exact definition of what the authors consider as open data, they refer to another publication. As the article should be self-contained, I would like to see the definition in the paper (at least the most interesting parts).

We agree that an overview of the criteria for available datasets is necessary, and we have added that to the manuscript. More detail than what we added would seem to us beyond the scope of this article, but we state more clearly now why the definition is complex, and how it is covered in Bobrov et al., 2023.

- The article states "... a rater could miss a specific dataset, in which case IRR was calculated only on those datasets extracted by all raters". If I understand this correctly, this introduces a bias in the evaluation, as datasets that where not identified by a rater a skipped during evaluation. When applying the final workflow with only one rater, those datasets would be missing entirely.

	Old IRR	Nr. of datasets (old)	New IRR	Nr. of datasets (new)
3 raters (dataset level)	0.762	26	0.793	31
2 raters (dataset level)	0.819	146	0.81	169
3 raters (article level)	0.826	20	0.826	20
2 raters (articles level)	0.901	100	0.901	100

We agree that this for an overall assessment of the reliability of results, the measure suggested by the reviewer is better. We have thus additionally calculated the IRR as suggested. The numbers in the table above indicate that if we explicitly consider datasets only detected by some raters, but not others, this does not substantially change the IRR. On the contrary, for three raters the IRR value even increased (see (iv) for more details). For two raters and a larger set of extractions, the IRR decreased very slightly from 0.819 to 0.810. We have added information on this analysis to the methods, and now report numbers on the "new" way of analyzing dataset-level IRR in the results.

It is an important point whether there is the possibility for those cases where only one rater screened an article to have misclassified it altogether, or what the probability of such an event is. In addition to the change in IRR after recalculation being only minor, several other lines of

reasoning support that the probability of completely misclassifying an article (i.e., classifying it as “open data” where this is not the case, and vice versa) is very low:

(i) In the 100 articles extracted independently by two raters, it was never the case that one rater detected an available dataset, the other did not, and the final assessment was that there was indeed an available dataset; this case would have allowed for a false negative, but it never occurred; we have added this information to the discussion section of the article
(ii) In the same 100 article, the reverse case occurred only once, where one rater detected an available dataset, the other rater did not detect it, and the overall assessment was that the dataset was not an available dataset by our definition; this case could have allowed for a false positive
(iii) Importantly, the case described under (ii) was a rare case of data underlying a systematic review, and when consulting an independent expert on systematic review, the initial assessment of the case did not agree with our final assessment; this indicates that the case is a borderline case, while also showing the limits in precision, especially if the criteria described in Bobrov et al. (2023) are not applied meticulously
(iv) For the 20 articles screened by three raters, three datasets were not detected by one of the raters, and one dataset was not detected by two out of three raters. All of these datasets were so-called “source data”. These are supplementary data and were ultimately never considered open data, but due to our decision to exclude supplementary data, some raters did not even begin to extract them. While this indicates degrees of flexibility in the exact procedure, this has no impact on the overall assessment, and indicates that the cases which differed between raters can typically be easily avoided.
(v) Lastly, the probability of missing a dataset can be expected to be inversely correlated to the number of datasets extracted per article. If ODDPub flagged an article, the rater can be expected to look for an available dataset very thoroughly. However, ODDPub does not indicate the number of available datasets, and it is very probably easier to miss additional datasets.

- When it comes to the interpretation of the IRR, the authors argue that maximizing the IRR was not focus of the study but the generation of reliable data. This should be the default case to estimate the quality. Later, the authors further argue that using the class category "unsure" less liberally, would further increase the IRR. I don't understand this argumentation, as the final goal is to get all important information from the articles and not optimizing the IRR. The analysis of the IRR reads a bit like "we could have cheated, but we didn't"

Even though this is surely not what we wanted to communicate, we understand why our point did not come across well. Definitely improving the information was and must be the goal. However, there are different levels of information at play, which are potentially in conflict: the individual assessment level, and the overall assessment level. We wanted to point out here that we accepted a lower precision of the individual decision to channel these cases into the reconciliation procedure and thus ultimately increase the precision of the overall decision. We have adjusted the manuscript accordingly.

- In the interpretation of the results, it is argued that the high reliability is not only supported by the high IRR, but by an increased correlation of another study that employed the same protocol. In general, correlation could also be increased by additional errors, thus the authors should elaborate on this and state in how far this supports their results.

We are not fully sure whether we understand this comment. It is of course conceivable that there are systematic errors, but these would not be exacerbated by conducting a second,

independent study. The correlation we refer to is fully within the study by Haven et al. (2023), and is not a correlation between their dataset and the Charité datasets. Regarding the extraction procedure, we would also like to mention that the Haven et al. dataset and the Charité datasets (2020 and 2021) have been created by different raters, thus decreasing the probability of biases due to person-specific procedures.

- The results of Krippendorffs alpha is interpreted by the approach of Landis&Koch 1977, who base their work on Cohen's kappa. Is it reliable to do this generalization?

Thank you for pointing this out. We are not sure whether such a generalization is indeed justified, and we now refer to Krippendorff (2004) as a reference for the interpretation of Krippendorffs alpha. The interpretation of an IRR >0.8 by Krippendorff (2004) as "reliable", seems, at least in its wording, "very conservative" according to Dobbrunz et al., 2021[1]. In addition, some authors refer to an agreement with Krippendorff's alpha values >0.8 as "excellent" (El-Tawil et al., 2019[2]). They refer to Landis & Koch (1977), as we did, but might not have been aware of the question on generalization. However, all of this seems to be too detailed to discuss in the article, and we now refer to Krippendorff's wording only.

- When evaluating the duration of the manual screening, the authors estimate 4.5 minutes per article. This shows that the process does not scale well. Further, the authors state that actual time to spend is much higher due to unsure cases. I'm puzzled by this statement. What is the purpose of the estimation, when the authors do not believe the result. Also, while the authors state that 4 minutes seems to be low, I have the feeling that it is rather high just to check if an identified dataset is publicly available or not. Please elaborate!

We have made an adjustment to the text which hopefully resolves the issue that it sounded as if we did not believe our results. Maybe this is partly due to the term "unsure", which can occur in assessments on the individual level. However, it cannot occur in the final assessment of data availability.

The 4.5 minutes are not an estimation but actually a value we measured. Of course, whether this is to be considered low is subjective. We think that in a discussion section, such subjectivity is generally justified, but we agree that we might have made the point too strongly, and we thus changed the wording from "very" to "quite". The point we wanted to stress here is that it might sound as if it could be done in a week for a whole institution, but actually this is not the case. Thus, we do agree that the process does not scale well to the national level or even the whole body of literature. We do not claim that it is, and other screening processes like the one implemented by the French Open Science Monitor using the DataStet tool are much better suited for this scale. The strength of our approach lies in its precision, which is needed for our specific purpose.

If checking the presence of a dataset only required finding the data availability statement and clicking on a link provided there to see whether the link resolves, this would surely last below a minute. However, there are multiple criteria we check to determine whether a dataset is available by our definition. We hope that the list of criteria we have now included makes this

[1] Dobbrunz S, Brunner F, Müller JL, Briken P. Interrater-Reliabilität der kriteriengeleiteten Beurteilung der Schuldfähigkeit bei paraphilen Störungen [Interrater reliability of the criteria-based assessment of criminal responsibility in paraphilic disorders]. Nervenarzt. 2021 Jan;92(1):1-8. German. doi: 10.1007/s00115-020-00920-1. PMID: 32394005; PMCID: PMC7808990.

[2] El-Tawil, S., Mair, G., Huang, X., Sakka, E., Palmer, J., Ford, I., Kalra, L., Wardlaw, J., Muir, K. W., & Study Collaborators (2019). Observer Agreement on Computed Tomography Perfusion Imaging in Acute Ischemic Stroke. *Stroke*, *50*(11), 3108–3114. https://doi.org/10.1161/STROKEAHA.119.026238

more transparent. In addition, for the resolution of disagreements, it is necessary to document **why** the assessment was made in a specific way.

- The novel parts of the process are mainly based on manual work. It would be nice if the authors outline in how far the entire process could be automated to eventually apply the incentivization on a regular basis.

We do already apply the incentivization for some years, and even though the process is time-consuming, it is established and works reasonably well for us. That said, we do of course look out for more automatization, and maybe we will ourselves use another tool in the future, if it shows to be more precise than ODDPub. Of course, full automatization would be ideal, but so far obituaries and corrigenda, we do not see a way to make it sufficiently reliable for our purpose of institutional incentivization. We do, however, refer in the outlook session to other tools which are intended to be applied without further manual screening, and have added further text addressing this.

- Figure 1 is missing the articles that could actually be retrieved due to closed or restricted access. Further, in order to make a statement about potential application in other setting it might be interesting to get an idea of the amount of open access articles in comparison with others.

In the methods we stated that "access to the article full texts is a prerequisite and thus, depending on institutional access rights, a substantial portion of articles might be excluded from analysis.". However, this is not the only reason for not downloading articles, and reporting the number of articles missed specifically due to restricted access would require extensive further analysis. There are two further reasons why some articles were not downloaded and screened with ODDPub: (i) we deliberately excluded articles of some types as e.g. obituaries and corrigenda to reduce the computational workload, and (ii) in some cases even articles which were open access or for which we had institutional access could not be downloaded automatedly due to barriers against automated tools placed by the journals or publishers. We have added the inclusion and exclusion criteria applied in the creation of the article sample, as well as a mention of download limitations, to the methods section. To find the requested number we would have to download all of the 6000+ articles again and investigate the reasons why each article was not downloaded. Apart from the fact that the availability of articles changes over time, and we could thus in any case not reproduce the exact numbers from the articles, this would be a large effort which we hope is not expected in this case. Thus, we did not implement the suggestion to add to the figures the number of articles which were not retrieved due to restricted accessed.

What we do know, however, is the share of open access publications from our institution, as reported in the QUEST Dashboard on Responsible Research. Out of all articles in 2021, 72.5% were either green or gold open access, including articles in hybrid journals. We have also added in the text, based on the comment of another reviewer, a comparison of data availability rates between open access and restricted access articles. For the reasons described above, this number is an approximation, as we did not screen some articles, and the share of open and restricted-access articles amongst these is unknown.

Beside the issues listed above, I see a couple of technical issues that could easily be fixed. (The order does not reflect importance):

- In the abstract, the authors state that an IRR of >0.8 was estimated, but there is no information about what kind of measure was used for estimation.

We have added the information in the abstract that the measure of IRR used is Krippendorff's alpha.

- The KrippAlpha function from the DescTools package was used. The function was cited, but not the packages. I suggest to cite the package directly.

Thanks for the recommendation, we have now cited the packages.

- The authors cite all software that was used in the study and use links to archived versions. However, neither a software version, nor the data of access was provided. While it would of course be possible to identify the software via software heritage, software citations should be accompanied by version (or dates).

- It might be good citation style to cite websites and software without date, I have the feeling that it would help the reader to add a date.

For software, we have added the version used, and in the case of ODDPub we now refer to the persistent version deposited in Zenodo. Where software was cited without having been used (e.g. DataStet), we have added the date of website access. We have also tried to reopen all other websites and have updated the date of access in the references, except where the website is not available anymore.

- The link to the dataset published with the study is wrong, as it points to some sharepoint folder

Thanks for pointing it out, we have now fixed the link.

- When estimating the amount of open data the article states "... that 7.9% of articles had underlying openly available data. In addition, 1.05% of articles had shared restricted-access datasets, resulting in an overall 8.4% of articles for which at least one dataset was available". I would guess that the final value should be 8.95%. Please correct!

8.4% is indeed the correct number. We have made edits to the text and added the following sentence to avoid this misunderstanding: "As for a given article both open and restricted-access datasets could be available, the overall percentage is not the sum of both availability types (open and restricted)."

- There are some issues with the bibliography:

- Bobrov et al (2023) is regularly cited but not provided in the list of references

Thanks for pointing this out, that's of course very important as a reference. The preprint we now list has been accepted for publication recently, and if it appears in print soon, we will update the reference.

- Haven et al., 2023 is cited but in the list of references provided as "Haven, T. L., Abunijela, S., & Hildebrand, N. (2022, September 15). Biomedical supervisors' role modeling of responsible research practices: a cross-sectional study."

There was a mistake here, which we have now fixed by referring to the reviewed article from 2023, rather than the preprint from 2022.

- Serghiou et al. (2021) is missing in the references

We have added the missing reference.
